# Comparison of two manual therapy techniques in patients with carpal tunnel syndrome: A randomized clinical trial

**DOI:** 10.22088/cjim.11.2.163

**Published:** 2020

**Authors:** Ghadam Ali Talebi, Payam Saadat, Yahya Javadian, Mohammad Taghipour

**Affiliations:** 1 Mobility Impairment Research Center, Health Research Institute, Babol University of Medical Sciences, Babol, Iran

**Keywords:** Carpal tunnel syndrome, Mechanical interface mobilization, Nerve mobilization

## Abstract

**Background:**

Manual therapy techniques are part of physiotherapy treatment of carpal tunnel syndrome (CTS) which are classified into two groups including nerve mobilization and mechanical interface mobilization. The aim of the study was to find which manual therapy method-technique directed to mechanical interface and nerve mobilization–has superior beneficial effects on clinical and electrophysiological findings in conservative management of patients with CTS.

**Methods:**

Thirty patients with CTS participated into two groups namely: mechanical interface and nerve mobilization in this randomized clinical trial. The intervention was performed three times weekly for 4 weeks. Mechanical interface mobilization was directed to structures around the median nerve at the forearm and wrist. Techniques of median nerve gliding and tension were used in the nerve mobilization group. The outcome measures included visual analogue scale (VAS), symptom severity scale (SSS), hand functional status scale (FSS) and motor and sensory distal latencies of median nerve. Paired t-test and ANCOVA were used for statistical analysis.

**Results:**

At the end of the 4^th^ week of the treatment, the mean of VAS, SSS and FSS significantly improved in both groups (p<0.05), but the difference was not significant between the two groups (P>0.05). Although the mean of motor and sensory distal latencies of median nerve at the end of the treatment period only improved in the nerve mobilization group (p<0.05), the difference was not significant between the two groups (P>0.05).

**Conclusion:**

Mechanical interface mobilization and nerve mobilization techniques are not superior to each other in reducing pain and improving hand symptoms and functional status.

Carpal tunnel syndrome (CTS), the most common *peripheral neuropathy* in the upper limb occurs due to the entrapment of the median nerve at the wrist. CTS is diagnosed based on a series of clinical findings, including sensory problems in the sensory distribution of median nerve in the hand (first 3 digits), positive phalen's test, weakness and atrophy of the thenar muscle and electrophysiological findings (prolonged motor and sensory distal latencies of median nerve) (1). Frequent activities of the wrist and fingers or maintaining prolonged awkward postures of the wrists are the most common occupational risk factors in CTS. Other non-occupational factors include tenosynovitis of flexors of fingers, thickened transverse carpal ligament, fracture or dislocation of the distal radius or lunate, rheumatoid arthritis, lipoma, diabetes, hyperthyroidism and pregnancy (1-3). 

Some authors have stated that conservative treatments should be considered as the first treatment method for patients with mild to moderate CTS (3-6).

In addition, a large number of CTS patients try to avoid surgery and want to find other therapies (7). So, research is needed to find the best non-invasive methods for treatment of CTS. The use of manual therapy and therapeutic exercise techniques is considered part of the conservative treatments of CTS and beneficial effects of these methods have been reported in some studies (8-19).

A complete and comprehensive treatment should focus on the dysfunction of the mechanical structures around the nerve and nerve itself (20, 21). Certainly, mechanical structures around the median nerve at the wrist (such as the transverse carpal ligament, flexor tendons in the carpal tunnel, dimensions of the tunnel and adjacent bones) as well as the structures surrounding the nerve in more proximal regions of the limb should be considered in the pathomechanism of the CTS (20). Hence, the manual therapy methods, including carpal bone mobilization, stretching of the transvers carpal ligament, soft tissue release and gliding of flexor tendons are directed toward mechanical interfaces to remove the pressure around the nerve (20).

Normally, the peripheral nerves have a capacity for gliding and tensioning during the different positions and movements of the limbs (22-25). Studies have shown that the gliding ability of the median nerve is reduced in patients with CTS, and the normal tension capacity is adversely affected by neurodynamic maneuvers (26). Accordingly, a series of exercise and manual therapy techniques designed to address the nerve itself and normalize neurodynamic movement may help to alleviate CTS symptoms (20-22). Some sources proposed that mechanical interface structures (muscle, fascia and joint) should be firstly considered in clinical treatments, and if patients have persistent symptoms, then the nerve mobilization techniques should be applied (22). Based on Shacklock’s opinion, appropriate nerve mobilization techniques can be initiated at the beginning of the treatment by observing a number of considerations and precautions (20).

In the studies addressing the effects of manual or exercise therapy techniques in the management of CTS (8-19), a combination of techniques (both related to mechanical interface and nerve mobilization) has been used, and the isolated effects of each method have not been determined. Therefore, it is unclear which group of manual therapy techniques has better effects on patients with CTS. The aim of this study was to compare the effects of two manual therapy techniques, including techniques for mechanical interface and nerve mobilization on visual analogue scale (VAS), symptom severity scale (SSS), functional status scale (FSS) and findings of neural conductivity in patients with CTS.

## Methods

**Design and Participants**: The study was a randomized clinical trial with a two-group parallel design, conducted in Iran. The necessary sample size was calculated based on our previous study (11). To determine the sample size we use the VAS. Calculation of sample size was based on an alpha of 0.05 and a statistical power of 0.8. Patients were entered into the study based on positive findings in the clinical examination (complaints of pain, numbness or tingling in the first three digits for 6 months, positive phalen's sign) and on electro-diagnostic findings (sensory median nerve conduction velocity <40 m/s and *median motor distal latency>* *4*.2 msec.) (27). A total of 57 patients referred to Ayatollah Rouhani Educational and Therapeutic Center Babol City for the intervention of which 18 patients were excluded since they did not meet the inclusion criteria. Since the severe and very severe CTS patients need surgical procedure, so thirty nine patients aged 30-50 years with mild to moderate CTS began the study. 

However, 9 patients failed to complete all the outcome measures yielding 30 patients in the final analysis. Exclusion criteria were patients with median nerve involvement in proximal areas such as thoracic outlet syndrome, cervical radiculopathy, *a history of carpal tunnel release surgery, *steroid injection in the carpal tunnel, thenar muscle atrophy, and metabolic diseases such as diabetes, severe thyroid disorders, anemia and pregnancy (11). Only patients with mild to moderate CTS were entered into the present study according to the classification of *the American Association of Electrodiagnostic Medicine (*26). This association categorizes the severity of CTS into: 

1) Mild (sensory conduction velocity is slow on finger-wrist, but the distal motor latency is normal); 

2) Moderate (sensory conduction velocity is slow on finger-wrist, but the distal motor latency is increased);

3) Severe (sensory response is absent on finger-wrist, and the distal motor latency is increased) and 

4) Very severe (absence of thenar motor response).

The subjects participated in the current study after voluntary completion of the consent form approved by the Ethics Committee of Babol University of Medical Sciences with code no: MUBABOL.REC.1394.103. This study was registered at the Iranian Registry of Clinical Trials (IRCT) with the number of 201508182851N4.

**Figure 1:**
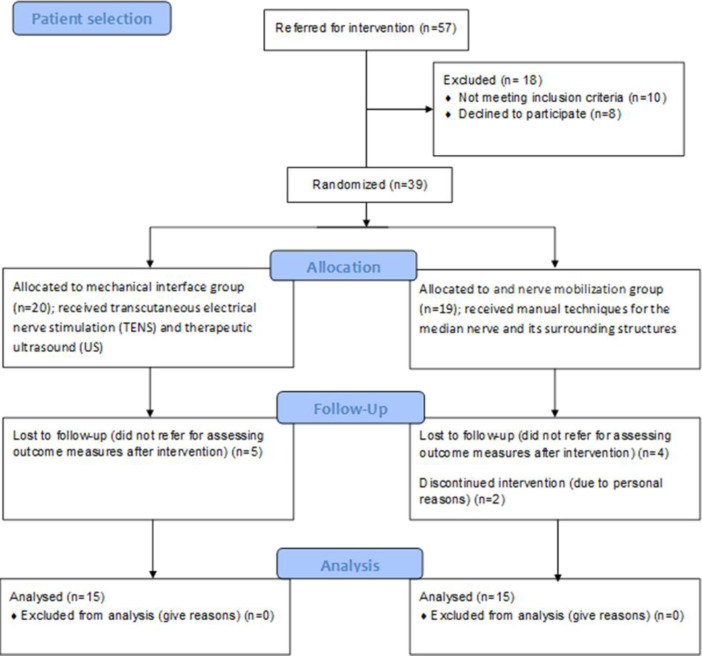
Flow diagram of phases through clinical trial

**Grouping and Interventions** **: **In total, 57 subjects participated in the study. Of these 57 subjects, 18 were excluded because they did not meet the inclusion criteria. The remaining participants were randomly allocated to mechanical interface (n=20) and nerve mobilization (n=19). Randomization was carried out by a simple random allocation (figure 1). 

**Figure 2:**
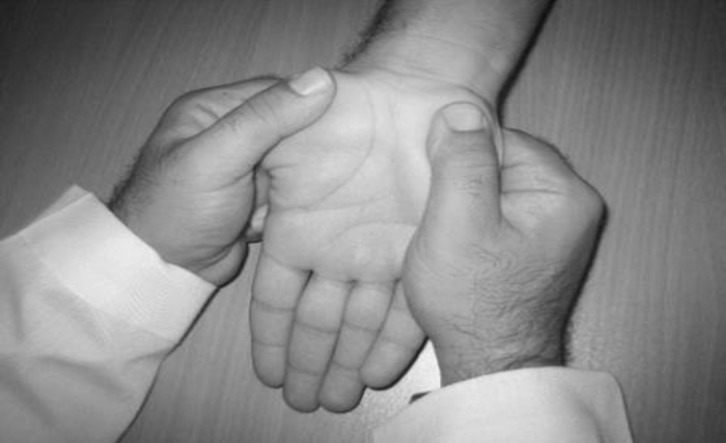
Transverse carpal ligament release

**Figure 3:**
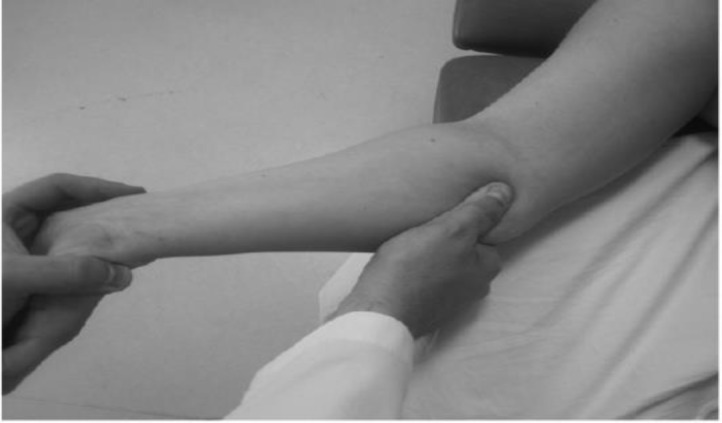
Soft tissue manipulation of the pronator teres19

Patients were alternately assigned to a group as they were identified. The participants were blinded for both grouping and treatment methods. The examiner collecting the outcome measures before and after treatment procedures and the data analyst were unaware of the assigned treatment. The interventions were performed three times a week for 4 weeks. In the mechanical interface group, five techniques, including wrist distraction (3 sets for 3 minutes), rhythmic and gentle stretching of the transverse carpal ligaments (figure 2), release of palmar hand fascia, gliding of the finger flexor tendons (using oscillatory flexion- extension movement of metacarpophalangial joint) and release of the upper forearm muscle and fascia (figure 3) were applied. Manual techniques were performed totally 15 minutes in each session that each technique included 3 sets for 3 minutes. 

To release the upper forearm muscle as demonstrated for pronator teres muscle in fig. 2, the therapist applied a firm pressure on the origin of the muscle by one thumb and concurrently moved the forearm into extension and supination (19, 20). 

**Figure 4:**
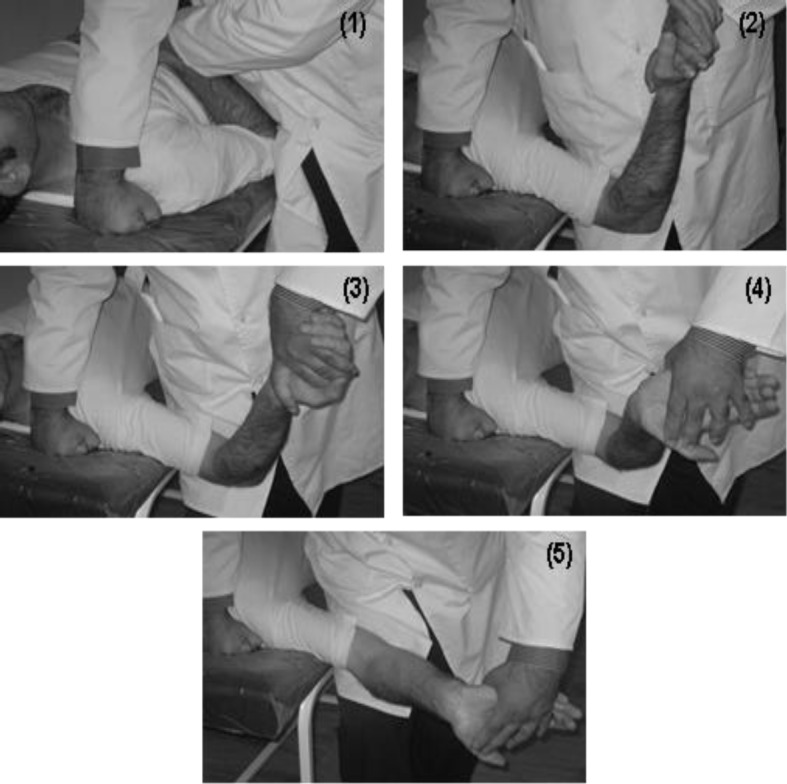
Stages of median nerve neurodynamic testing

In the nerve mobilization group, special techniques of median nerve mobilization include gliding and tension maneuvers with duration of 15 minutes in each session, were used (figure 4) (19, 20). 

The parameters of these techniques were determined and progressed based on the findings of the initial examination and the degree of CTS irritability during the treatment. A skilled and experienced physiotherapist in both groups applied the manual therapy techniques. At the beginning of each treatment session, both groups received therapeutic ultrasound (frequency of 1 MHz, intensity of 1 W/cm^2^, for 4 minutes) and transcutaneous electrical nerve stimulation (TENS) (frequency of 80 Hz, pulse duration of 60 µs, at the level of comfortable tingling sensation, for 20 minutes).

**Outcome Measures: **VAS (11), Boston questionnaire (containing symptom severity scale (SSS) and functional severity scale (FSS) (28) and distal latency of median nerve (1, 27) were evaluated before and immediately after the end of the treatment period. The distal latency of median nerve was evaluated by a neurologist and other outcome measures were assessed by a physical therapist.

**VAS: **A visual analogue scale (VAS) via 11-point numerical pain rating scale (0=no pain to 10=maximum pain) was used to assess the current level of pain and hand discomfort (11).

**Boston Questionnaire: **The Boston Questionnaire is a standardized, patient-based outcome measure of symptom severity and functional status in patients with carpal tunnel syndrome (28). The questionnaire including two parts, namely the symptom severity scale (SSS) and the functional status scale (FSS), is considered a standard tool to evaluate the patients with CTS (29). The SSS contains 11 questions on different symptoms of hand and FSS comprises of 5 questions assessing the difficulty in performing selected activities. The response to each question was scored from one (mildest) to five (most severe) points. The overall scores for SSS and FSS were calculated as the score sum of all questions.

**Distal latency of Median Nerve: **Distal sensory latency (milliseconds; DSL) of median nerve was measured in its standard manner, in which the examined wrist was stimulated, and the peak latency was recorded 14 cm away in the middle finger. Distal motor latency (milliseconds; DML) of median nerve was measured from the wrist to the abductor pollicis brevis muscle (1, 27). 

**Data Analysis: **Data were analyzed using SPSS Version 24. The Kolmogorov-Smirnov test was used to verify the normal distribution of data. Paired t-test and ANCOVA were applied to compare the data in each group and between the two groups, respectively. A p<0.05 was considered significant level.

## Results

Thirty patients with CTS (mean age=50 years, mean weight=77 Kg and mean duration of hand symptoms=29 months) participated in the current study. Based on Kolmogorov-Smirnov test, all variables, including VAS, SSS, FSS, DSL and DML and demographic variables containing age, weight and duration of CTS had normal distribution. According to the independent T-test, there were no significant differences between the two groups (mechanical interface and nerve mobilization) in any of the variables at baseline (p>0.05) (table 1). In the group of mechanical interface, paired t-test revealed that the mean of VAS (P<0.001), SSS (P<0.001) and FSS (P=0.001) improved significantly, but the mean of DSL (P=0.148) and DML (P=0.063) had no significant improvement at the end of the treatment period (table 2). Based on paired t-test, the mean of VAS (P<0.001), SSS (P<0.001), FSS (P=0.001), and DSL (P=0.001) and DML (P=0.036) significantly improved in the nerve mobilization group at the end of the treatment period (table 2). Moreover, ANCOVA test indicated that there was no significant difference between the two groups in VAS (P=0.810), SSS (P=0.130), FSS (P=0.420), DSL (P=0.230) and DML (P=0.530) at the end of the treatment period (P>0.05) (table 3 and fig. 4).

**Table1:** Patient’s characteristics at baseline

**Group**	**Interface Mobilization** **(N= 15)**	**Nerve Mobilization** **(N=15)**	**p-value**
Age (years)	48.86 ± 8.94	51.46 ± 9.62	0.450
Weight (Kg.)	76.86 ± 10.58	78.13 ± 16.44	0.804
Duration of hand symptoms (Month)	30.46 ± 22.90	29.06 ± 28.00	0.882
VAS	6.80 ± 1.65	6.40 ± 1.45	0.488
SSS	30.13 ± 8.95	30.66 ± 7.82	0.863
FSS	19.33 ± 8.05	17.20 ± 6.77	0.439
SDL (msec.)	6.39 ± 2.73	6.22 ± 1.65	0.833
MDL (msec.)	6.18 ± 1.65	6.26 ± 1.8	0.898

VAS: visual analogue scale; SSS: symptom severity scale; FSS: functional status scale; SDL: Sensory Distal Latency; MDL: Motor Distal Latency

**Table2:** Comparison of variables, before and after the intervention within the groups

**Group**	**Interface Mobilization**			**Nerve Mobilization**		
	**Mean ± SD** **Before**	**Mean ± SD** **after**	**P value**	**Mean ± SD** **before**	**Mean ± SD** **after**	**P value**
VAS	6.80 ± 1.65	3.93± 1.90	0.000	6.40 ± 1.45	3.53 ± 2.23	0.000
SSS	30.13± 8.95	21.73± 8.22	0.000	30.66 ± 7.82	19.26 ± 5.48	0.000
FSS	19.33 ± 8.05	14.53 ± 5.13	0.001	17.20 ± 6.77	12.33 ± 5.48	0.001
SDL	6.39 ± 2.73	5.39 ± 1.19	0.148	6.22 ± 1.65	5.85 ± 1.68	0.001
MDL	6.18 ± 1.65	5.76 ± 1.15	0.226	6.26 ± 1.85	5.60 ± 1.40	0.036

**Table3:** Comparison of mean difference of the variables between the two groups at the end of 4th weeks

**Group**	**Interface Mobilization** ** Mean ± SD**	**Nerve Mobilization** ** Mean ± SD**	**F**	**df**	**P value **
VAS	2.86 ± 2.06	2.86 ± 1.88	0.06	1	0.81
SSS	8.40 ± 4.79	11.40 ± 6.76	2.42	1	0.13
FSS	4.80 ± 4.29	4.86 ± 4.64	0.67	1	0.42
SDL	1.00 ± 2.52	0.36 ± 0.35	1.48	1	0.23
MDL	0.42 ± 1.28	0.66 ± 1.11	0.40	1	0.53

**Figure 5:**
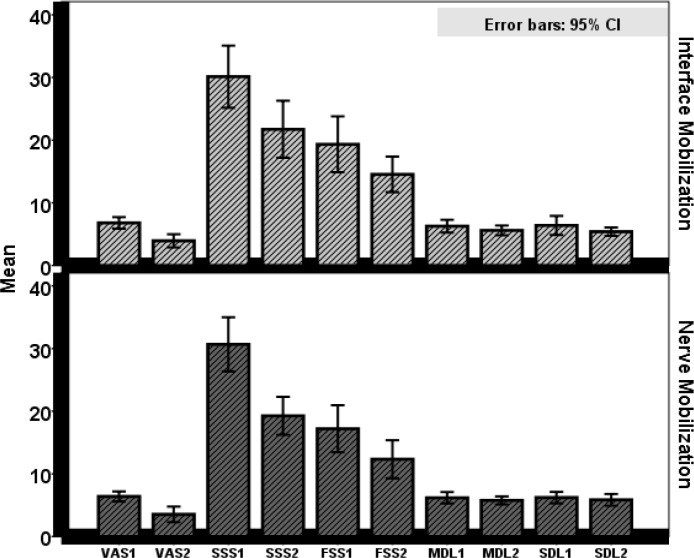
Bar diagram representing comparison of outcome measures before and after intervention at both groups

## Discussion

The results of the present study indicated that both manual therapy techniques directed to mechanical interface of median nerve and nerve mobilization for 4 weeks led to significant reduction of the pain severity and significant improvement of SSS and FSS in patients with CTS, with no difference noted between groups. Significant effects on electrodiagnostic parameters (sensory and motor latencies of median nerve) only occurred in the nerve mobilization group. Sensory and motor latencies of median nerve significantly improved at the end of the 4^th^ week in the nerve mobilization group and the difference between the two groups was not significant. Previous studies have demonstrated that a combination of manual and nerve mobilization techniques have positive and beneficial effects on improving clinical symptoms in CTS patients (8-19). 

*Akalin* et al. observed a significant improvement in some clinical symptoms and tests in CTS patients by using splint, nerve and tendon gliding exercise (8). In addition, Pinar et al. (9) reported a similar result by using splint and median nerve gliding techniques. Seradge et al. (10) have argued that the intermittent active exercise of wrist and finger for one minute can decrease the pressure *inside the carpal tunnel*. 

Oskouei et al. indicated that 4-week manual therapy, including stretching of *the flexor retinaculum* and transverse carpal ligaments, tendon gliding techniques and median nerve mobilization, along with physiotherapy modalities, ultrasound and TENS, caused a significant improvement of VAS, SSS, FSS, median neurodynamic test and sensory latency of median nerve in patients with CTS (11). Bongi et al. reported that using 3-week manual therapy techniques, including wrist and hand soft tissue release and carpal bone mobilization significantly improved the hand symptoms and functions (based on Boston's questionnaire) and reduced paresthesia, pain and hand sensitivity but with no significant effects on neural conductivity (12).

In another study, Burke et al. reported that 4-week manual therapy consisting of soft tissue mobilization through deep manual pressure on scar tissue and tight muscle, stretching the connective tissues and fascia of the hand, wrist and forearm significantly improved the motor and sensory latencies of median nerve, VAS, SSS, FSS, grip *strength and wrist range of motion* in patients with mild to moderate CTS (13).

According to Rincon et al., the use of one session of soft tissue mobilization (for 30 minutes) and median nerve* gliding techniques *(for 5 to 10 minutes in 2 sets) significantly reduces the hand pain intensity in patients with CTS, while had no significant effect on the sensitivity of pressure pain in different regions (14). Still, the exact mechanism of effectiveness of manual therapy is not clear. The mechanical and neurophysiologic mechanisms are likely to be involved. One theory is that manual therapy affects several central mechanisms of pain control, including descending pain inhibitory mechanisms, especially in the periaqueductal grays (30- 32). Some studies referred to hypoalgesic effects of neurodynamic techniques (31).

 The effectiveness of neuromobilization techniques seems to be multifactorial and may be due to (a) decreased the endoneurial pressure in *the carpal tunnel* and decreased tissue edema consequently minimize the nerve hypoxia and pain symptoms (7,20, 22) (b) produced an environmental stimulus eliminating the sensitization process. In addition, the activation of descending *inhibitory* pathways may be involved in this regard (30- 32). Also, Wolny T Linek P believed that the use of neurodynamic techniques may increase blood supply, reduce mechanical irritation and improve nerve sliding to improve its physiological function, that is, to reduce intraneural edema, improve axonal transport, and decrease intraneural pressure, thereby reducing mechanical sensitivity (7). Shacklock (20) claimed that when a therapeutic package is designed for a neurodynamic problem, therapeutic techniques should also focus on both mechanical interface and nerve structures. The findings of this study demonstrated that each of the manual therapy methods, including mechanical interface mobilization and nerve mobilization, in turn, could reduce the severity of the hand symptoms and functional status, but there was no significant difference between the two methods. 

We think insignificant difference of sensory and motor latencies of median nerve between the two groups could be attributed to low sample size as the main limitation of this work. Another limitation is the use of electrophysical modalities including US and TENS in both groups. The limitation here is that the improvement of the hand symptoms, functional status and pain severity may be due to non-specific effects of such modalities. 

Although we should ethically use a standard conservative protocol for CTS patients. An additional research limitation is that the study rated only the short-term outcomes. Therefore, we proposed further research by a larger number of patients and follow-up to understand the therapeutic effects of mechanical interface mobilization and specific neurodynamic techniques.

The main strength of our study is the clear and understandable methodology for both the diagnosis and treatment of patients with carpal tunnel syndrome. Diagnostic criteria were comprehensive and included interview, functional tests, and nerve conduction study. The treatment protocol of both manual techniques including mechanical interface mobilization and neurodynamic techniques were described in detail, so it can be easily used in clinical practice by clinicians and also repeated by researchers in future studies. 

In conclusion, the use of manual therapy methods, including mobilization of the mechanical interface and specific neurodynamic techniques in conservative treatment of mild to moderate forms of carpal tunnel syndrome has significant therapeutic benefits such as improvement in hand symptoms and functional status as well as reduction of pain. In addition, improvement in nerve conduction by specific neurodynamic techniques emerged. 

Finally, these two manual techniques are not superior to each other in reducing pain and improving in hand symptoms and functional status. In further studies, it could be worthwhile to evaluate the effectiveness of manual techniques and compare it with other physiotherapy methods/ techniques such as exercise therapy or electrophysical modalities. In addition, we believe that future studies should compare sliding and tensioning neurodynamic techniques to get information about their separate treatment potentials.

## Funding:

This research study was founded by the yia chancellery for research and technology of Babol University of Medical Sciences.
